# Assessment of Serum Thyroid Antibody Levels in Patients with Type-I and Type-II Diabetes without Thyroid Disease

**DOI:** 10.12669/pjms.41.7.11918

**Published:** 2025-07

**Authors:** Akin Dayan, Asli Karadeniz Yonak

**Affiliations:** 1 Akin Dayan Department of Family Medicine, Diabetes Polyclinic, Haydarpasa Numune Training and Research Hospital, Istanbul, Turkey; 2 Asli Karadeniz Yonak Department of Internal Medicine, Haydarpasa Numune Training and Research Hospital, Istanbul, Turkey

**Keywords:** Antibodies, Diabetes mellitus, Thyroglobulin, Thyroid peroxidase, Thyroiditis

## Abstract

**Objective:**

The study assessed differences in the thyroglobulin antibody and thyroid peroxidase antibody between euthyroid Type-I and Type-II diabetes mellitus (DM) and control groups not using thyroid medication.

**Methods:**

This was a cross-sectional study. People with Type-I and Type-II diabetes and a control group aged between 18 and 80 years were enrolled at the diabetes and internal medicine outpatient clinic of Istanbul Haydarpaşa Numune Training and Research Hospital between 2020-2023. Data analysis was performed on 103 participants with Type-I DM, 110 with Type-II DM, and 110 control subjects, all randomly selected. Anti-thyroglobulin antibody (anti-TG), anti-thyroperoxidase antibody (anti-TPO), thyroid-stimulating hormone (TSH), fasting blood glucose (FBG), hemoglobin A1c, (HbA1c) and blood creatinine levels were recorded for analysis.

**Results:**

In the study, anti-TG and anti-TPO levels were higher in Type-I DM patients than in the control and Type-II DM groups. This elevation was statistically significant in women with Type-I DM compared to women patients in other groups. In this study, thyroid antibody levels in patients with Type-II DM were not significantly higher than those in the control group.

**Conclusions:**

This study highlights the importance of regular assessment of thyroid function and antibodies in women diagnosed with Type-I DM, but not necessarily in those wi th Type-II DM without thyroid disease.

## INTRODUCTION

Thyroid disorders and DM are among the most common endocrine disorders. An elevated prevalence of chronic autoimmune thyroiditis (AIT) has been observed in patients with Type-I DM compared with that in the non-diabetic population.^1^,^2^ Although studies indicate a higher occurrence of thyroid disorders in Type-II DM than in the general population, it remains unclear whether the prevalence of euthyroid or hypothyroid AIT increases in Type-II DM.^3^-^5^

Hypothyroidism can lead to disturbances in metabolic control in patients with diabetes.^6^ Subclinical hypothyroidism is associated with symptomatic hypoglycemia in Type-I diabetes.^7^ Thyroid hormones stimulate intestinal glucose absorption, glycogenolysis, and hepatic insulin catabolism, leading to hyperglycemia. Mild alterations in thyroid hormone levels can increase the risk of hypoglycemia.^8^,^9^

Autoantibody positivity and autoimmune thyroid disease are more common in women, possibly influenced by sex hormones.^10^,^11^ Possible reasons for the difference between thyroid autoimmunity and sex differences include the protective effect of androgens against autoimmune progression and estradiol, which accelerate the progression of autoimmune diseases through T-lymphocyte.^12^ Studies investigating the prevalence of chronic autoimmune thyroid antibodies in euthyroid Type-I and Type-II DM, as well as controls in women and mensimultaneously, are scarce in the literature. The present study evaluated the impact of Type-I and Type-II DM on AIT markers, specifically anti-TG and anti-TPO markers, considering sex differences.

## METHODS

This retrospective cross-sectional study included patients with Type-I DM, Type-II DM, and nondiabetic controls. Participants were selected through simple random sampling from individuals referred over a 3-year period (2020-2023) to the Diabetes and Internal Medicine Outpatient Clinics at Istanbul Haydarpasa Numune Training and Research Hospital.

A total of 1,500 patients met the inclusion criteria. The minimum required sample size was calculated to be 306, with at least 102 participants in each of the three groups. Ultimately, 323 individuals were enrolled: 103 with Type-I DM, 110 with Type_II DM, and 110 controls.

### Etical Approval:

The study was approved by the Ethics Committee of Istanbul Haydarpasa Numune Training and Research Hospital (approval no. 2023/237. Date December 25, 2023). The study was conducted in accordance with the Declaration of Helsinki.

### Inclusion and exclusion criteria:

Patients aged 18-80 years with and without diabetes were included in the study. Subjects with a history of thyroid disease, use of thyroid medication, myocardial infarction within the previous six months, class 3 or 4 heart failure, use of medications that may interfere with metabolic control (such as synthetic glucocorticoids or immunosuppressive treatment), rheumatological disease, chronic liver disease, infectious disease, malignancy, and pregnant women were excluded from the study.

### Study design:

The subjects’ sex, age, duration of diabetes, total insulin dose, height, weight, body mass index (BMI), and laboratory results such as anti-TG, anti-TPO, TSH, FBG, HbA1c, and blood creatinine were recorded. The participants were categorized into three groups: control, Type-I DM, and Type-II DM. We also classified the samples based on thyroid antibody levels (high, i.e., above the upper limit, and normal) and by sex. TSH, anti-TG, and anti-TPO levels were quantified using the chemiluminescent microparticle immunological method, employing the Abbott Architect I 2000 SR® device (USA). The cutoff value for anti-TPO positivity was set at 5.0 IU/ml, whereas for anti-TG, it was 4.0 IU/ml. These values were determined according to the methods and kits used in our hospital. The normal range for TSH levels was established as 0.5-5.0 μU/ml by laboratory calibration guidelines.

### Statistical analysis:

Statistical analyses were performed using SPSS 20.0 for the Windows program. Categorical variables are presented as numbers and percentages, whereas numerical variables are presented using descriptive statistics, such as median and interquartile range (IQR). Distribution analysis was performed using histograms and the Shapiro-Wilk test. Intergroup categorical comparisons were performed using the chi-square test. Numerical comparisons were performed using the Kruskal-Wallis test for independent groups, for which this assumption was not met. Pairwise comparisons were adjusted using Bonferroni correction and were subjected to the Mann-Whitney U test. Logistic regression analysis was used to identify the factors predicting high anti-TG and anti-TPO antibody levels. The independent variables were based on previous studies and the current study population. The model included diabetes status (Type-I DM, Type-II DM), gender, age, BMI, TSH, HbA1c, and blood creatine parameters as independent variables. Statistical significance was set at p<0.05.

## RESULTS

AAmong the 323 participants, 34.1% (n = 110) were included in the control group, 31.8% (n = 103) had Type-I DM, and 34.1% (n = 110) had Type-II DM. The median age was 39 years (IQR: 27), and 69.3% (n = 224) were women. Demographic characteristics, physical examination findings, and laboratory results for each group are summarized in Table I.

**Table-I:** Demographic data, physical examination and laboratory findings in control and diabetic groups.

		All patients (n: 323)	Control 34.1%, (n:110)	Type-I DM 31.8%, (n:103)	Type-II DM 34.1%, (n:110)	p
Gender (%)	Women	69.3 (224)	83.6 (92)	53.4 (55)	70.0 (77)	<0.001*
	Men	30.7 (99)	16.4 (18)	46.6 (48)	30.0 (33)	
Age (year)	All	39.00 (27.00)	38.00 (19.25)^a, b^	28.00 (12.00)^a, c^	56.00 (10.50)^b, c^	<0.001**
	Women	41.00 (25.00)	37.00 (18.50)^a, b^	29.00 (13.00)^a,c^	56.00 (9.00)^b, c^	<0.001**
	Men	34.00 (34.00)	43.50 (24.75)^a,b^	25.00 (11.75)^a, c^	60.00 (18.00)^b, c^	<0.001**
Weight (kg)	All	71.00 (21.00)	68.00 (21.50)^a, b^	64.00(13.00)^a, c^	82.00 (22.25)^b, c^	0.008**
	Women	69.00 (21.00)	65.00 (20.75)^a, b^	62.00 (15.00)^a, c^	81.00 (18)^b, c^	<0.001**
	Men	75.00 (22.00)	77.50 (17.25)^a^	67.50 (12.50)^a, b^	88.00 (29.5)^b^	<0.001**
Height (cm)	All	160.00 (13.00)	160.50 (13.00)^a^	164.00 (14.00)^a, b^	157.00 (13.00)^b^	0.003**
	Women	157.00 (9.00)	158.50 (11.00)^a^	159.00 (7.00)^b^	155.00 (6.50)^a, b^	<0.001**
	Men	172.00 (11.00)	172.00 (13.00)	172.5 (9.00)	169.00 (10.50)	0.339**
BMI (kg/m^2^)	All	26.95 (9.26)	26.26 (7.13)^a, b^	23.01 (4.88)^a, c^	32,85 (6.69)^b, c^	<0.001**
	Women	27.98 (9.73)	26.66 (7.62)^a, b^	23.83 (5.29)^a, c^	33.33 (6.32)^b, c^	<0.001**
	Men	25.43 (7.60)	25.67 (7.16)^a,b^	22.72 (4.00)^a, c^	30.35 (8.20)^b, c^	<0.001**
Diabetes Duration (year)	All	10.00 (10.00)		9.00 (9.00)	10 (10)	0.107***
	Women	10.00 (09.00)		11.00 (8.00)	10.00 (9.50)	0.912***
	Men	8.00 (10.75)		7.00 (10.12)	10.00 (12.50)	0.102***
TSH (mIU/ml)	All	1.62 (1.20)	1.60 (1.09)	1.72 (1.44)	1.52 (1.14)	0.314**
	Women	1.14 (1.32)	1.60 (1.22)	1.93 (1.72)	1.62 (1.18)	0.262**
	Men	1.47 (1.00)	1.63 (0.78)	1.58 (1.20)	1.30 (092)	0.332**
Anti-TG Antibody (IU/ml)	All	0.90 (0.30)	0.90 (0.00)	0.90 (2.00)	0.90 (0.13)	0.088**
	Women	0.90 (1.08)	0.90 (0.18)^a^	0.90 (6.00)^a^	0.90 (0.45)	0.021**
	Men	0.90 (0.00)	0.90 (0.18)	0.90 (0.00)	0.90 (0.00)	0.685**
Anti-TPO Antibody (IU/ml)	All	0.80 (2.10)	0.80 (1.23)^a^	1.10 (10.30)^a^	0.80 (1.43)	0.031**
	Women	1.00 (4.18)	0.90 (1.20)^a^	2.60 (60.00)^a, b^	0.80 (1.60)^b^	0.002**
	Men	0.60 (1.30)	0.40 (0.75)	0.60 (1.48)	0.70 (1.45)	0.405**
FBG (mg/dl)	All	126.50 (93.75)	91.00 (10.25)^a, b^	226.50 (180.50)^a, c^	160.50 (97.25)^b, c^	<0.001**
	Women	117.50 (115.75)	91.00 (10.00)^a, b^	237.00 (156.00)^a, c^	164.00 (96.00)^b, c^	<0.001**
	Men	147.00 (149.5)	89.50 (11.25)^a, b^	189.00 (195.00)^a^	152.00 (123.50)^b^	<0.001**
HbA1c (%)	All	7.10 (3.20)	5.60 (0.50)^a, b^	9.00 (3.10)^a, c^	7.95 (2.40)^b, c^	<0.001**
	Women	6.65 (3.18)	5.60 (0.50)^a, b^	8.80 (3.40)^a^	8.20 (2.70)^b^	<0.001**
	Men	7.90 (2.90)	5.65 (0.60)^a, b^	9.20 (2.68)^a, c^	7.80 (1.40)^b, c^	<0.001**
Blood Creatinine (mg/dl)	All	0.78 (0.22)	0.77 (0.17)	0.77 (0.20)	0.84 (0.28)	0.696**
	Women	0.73 (0.17)	0.75 (0.16)	0.71 (0.13)	0.74 (0.26)	0.067**
	Men	0.91 (0.19)	0.97 (0.09)^a^	0.86 (0.18)^a, b^	0.92 (0.22)^b^	0.001**
Total Insulin Dose(units/day)	All	50.00 (31.25)		52.20 (29.00)	42.00 (32.00)	0.012***
	Women	50.00 (28.75)		50.00 (28.00)	50.00 (27.00)	0.522***
	Men	49.00 (39.25)		54.00 (32.50)	34.00 (28.25)	0.001***

* Chi-Square test,

** Kruskal Wallis ,

*** Mann Whitney U Test,

^abc^Same letters express significant difference between groups, Abbreviations: Type-I DM: Type-I diabetes mellitus, Type-II DM: Type-II diabetes mellitus, BMI: Body mass index, TSH: Thyroid stimulating hormone, Anti-TG: Anti-thyroglobulin, Anti-TPO: Anti-thyroid peroxidase,HbA1c: Hemoglobin A1c FBG: Fasting blood glucose.

The distributions of elevated anti-TG and anti-TPO levels across the groups, including sex-based comparisons, are shown in Tables II and III.

**Table-II:** Distribution of the anti-TG and anti-TPO levels according to control, Type-I and Type-II diabetes.

	Anti-TG Antibody, % (n)				Anti-TPO Antibody, % (n)			
	Normal	Elevated	x^2^	p	Normal	Elevated	x^2^	p
***TOTAL***								
Control	87.3 (96)	12.7 (14)	6.17	0.046	84.5 (93)	15.5 (17)	13.87	0.001
Type 1 DM	77.7 (80)	22.3 (23)			67 .0 (69)	33.0 (34)		
Type 2 DM	89.1 (98)	10.9 (12)			85.5 (94)	14.5 (16)		
Total	84.8 (274)	15.2 (49)			76.8 (179)	23.2 (54)		
***WOMEN***								
Control	85.9 (79)	14.1 (13)	10.48	0.005	82.6 (76)	17.4 (16)	18.16	<0.001
Type 1 DM	69.1 (38)	30.9 (17)			54.5 (30)	45.5 (25)		
Type 2 DM	89.6 (69)	10.4 (8)			83.1 (64)	16.9 (13)		
Total	83.0 (186)	17.0 (38)			75.9 (170)	24.1 (54)		
***MEN***								
Control	94.4 (17)	5.6 (1)	0.69	0.708	94.4 (17)	5.6 (1)	2.71	0.258
Type 1 DM	87.5 (42)	12.5 (6)			81.3 (39)	18.8 (9)		
Type 2 DM	87.9 (29)	12.1 (4)			90.9 (30)	9.1 (3)		
Total	88.9 (88)	11.1 (11)			86.9 (86)	13.1 (13)		

Chi-Square test, Abbreviations: Anti-TGA: Anti-thyroglobulin antibody, Anti-TPO: Anti-thyroid peroxidase antibodies, Type-I DM: Type-I diabetes mellitus, Type-II DM: Type-II diabetes mellitus.

**Table-III:** Distribution of the anti-TG and anti-TPO levels according to sex in control, Type-I and Type-II diabetes groups

	Anti-TG Antibody, % (n)				Anti-TPO Antibody, % (n)			
	Normal	Elevated	x2	p	Normal	Elevated	x2	p
***TOTAL***								
Women	83.0(186)	17.0 (38)	1.83	0.176	75.9 (170)	24.1 (54)	5.03	0.025
Men	88.9 (88)	11.1 (11)			86.9 (86)	13.1 (13)		
***CONTROL***								
Women	85.9 (79)	14.1 (13)	0.459*		82.6 (76)	17.4 (16)		0.297*
Men	94.4 (17)	5.6 (1)			94.4 (17)	5.6 (1)		
***Type 1 DM***								
Women	69.1 (38)	30.9 (17)	5.01	0.025	54.5 (30)	45.5 (25)	8.27	0.004
Men	87.5 (42)	12.5 (6)			81.3 (39)	18.8 (9)		
***Type 2 DM***								
Women	89.6 (69)	10.4 (8)	0.07	0.790	83.1 (64)	16.9 (13)	1.13	0.288
Men	87.9 (29)	12.1 (4)			90.9 (30)	9.1 (3)		

Chi-Square test,

* Fisher's Exact Test Abbreviations: Anti-TGA: Anti-thyroglobulin antibody, Anti-TPO: Anti-thyroid peroxidase antibodies, Type-I DM: Type-I diabetes mellitus, Type-II DM: Type-II diabetes mellitus.

The distribution of thyroid antibodies by sex in the patient and control groups is shown in Table-IV. The percentage of subjects with abnormal antithyroid autoantibodies is presented in Table-V. Logistic regression analysis was used to identify factors predicting elevated anti-TG and anti-TPO antibody levels in the generated models. The Nagelkerke R2 values for the elevated anti-TG and anti-TPO antibody levels were 0.060 and 0.133, respectively (Table-VI and VII).

**Table- IV:** The distribution of thyroid antibodies by sex in patients and control group.

Antibodies	Gender	All patients (n: 323)	Control 34.1%, (n:110)	Type-I DM 31.8%, (n:103)	Type-II DM 34.1%, (n:110)
Both antibodies normal	All	74.9 (242)	80.0 (88)	62.1 (64)	81.8 (90)
	Women	71.9 (161)	77.2 (71)	50.9 (28)	80.5 (62)
	Men	81.8 (81)	94.4 (17)	75.0 (36)	84.8 (28)
Only Anti-TPO high	All	9.9 (32)	7.3 (8)	15.5 (16)	7.3 (8)
	Women	11.2 (25)	8.7 (8)	18.2 (10)	9.1 (7)
	Men	7.1 (7)	0 (0)	12.5 (6)	3.0 (1)
Only Anti-TG high	All	4.3 (14)	4.5 (5)	4.9 (5)	3.6 (4)
	Women	4.0 (9)	5.4 (5)	3.6 (2)	2.6 (2)
	Men	5.1 (5)	0 (0)	6.3 (3)	6.1 (2)
Both antibodies high	All	10.8 (35)	8.2 (9)	17.5 (18)	7.3 (8)
	Women	12.9 (29)	8.7 (8)	27.3 (15)	7.8 (6)
	Men	6.1 (6)	5.6 (1)	6.3 (3)	6.1 (2)

***Abbreviations:*** Type-I DM: Type-I diabetes mellitus, Type-II DM: Type-II diabetes mellitus, Anti-TPO: Anti-thyroid peroxidase antibodies, Anti-TGA: Anti-thyroglobulin antibody.

**Table-V:** Distribution of subjects with abnormal anti-thyroid autoantibodies.

Antibodies	Gender	All patients (n: 81)	Control 27.2%, (n:22)	Type-I DM 48.2%, (n:39)	Type-II DM 24.7%, (n:20)
Only Anti-TPO high	All	39.5 (32)	36.4 (8)	41.0 (16)	40.0 (8)
	Women	39.7 (25)	38.1 (8)	37.0 (10)	46.7 (7)
	Men	38.9 (7)	0 (0)	50.0 (6)	20.0 (1)
Only Anti-TG Antibody high	All	17.3 (14)	22.7 (5)	12.8 (5)	20.0 (4)
	Women	14.3 (9)	23.8 (5)	7.4 (2)	13.3 (2)
	Men	27.8 (5)	0 (0)	25.0 (3)	40.0 (2)
Both antibodies high	All	43.2 (35)	40.9 (9)	46.2 (18)	40.0 (8)
	Women	46.0 (29)	38.1 (8)	55.6 (15)	40.0 (6)
	Men	33.3 (6)	100.0 (1)	25.0 (3)	40.0 (2)

***Abbreviations:*** Type-I DM: Type-I diabetes mellitus, Type-II DM: Type-II diabetes mellitus, Anti-TPO: Anti-thyroid peroxidase antibodies, Anti-TGA: Anti-thyroglobulin antibody.

**Table-VI:** Logistic regression analysis for predicting elevated anti-TGA levels.

	B	S.E.	Exp(B)	p	95% CI.for Exp(B)
Constant	-5.153	1.792	0.006	0.004	
Type-I DM	1.351	0.614	3.863	0.028	1.160-12.862
Type-II DM	0.399	0.566	1.490	0.482	0.491-4.520
Gender (Women)	0.934	0.443	2.543	0.035	1.066-6.066
Age	-0.003	0.017	0.997	0.852	0.965-1.030
BMI (kg/m^2^)	0.033	0.033	1.034	0.321	0.927-1.078
TSH (mIU/ml)	-0.025	0.171	1.397	0.882	0.697-1.364
HbA1c (%)	0.021	0.095	1.021	0.827	0.847-1.230
Blood kreatinine	1.502	1.264	4.491	0.235	0.377-53.519

Cox & Snell R Square: 0.034, Nagelkerke R Square: 0.060. Abbreviations: Anti-TGA: Anti-thyroglobulin antibody, Type-I DM: Type-I diabetes mellitus, Type-II DM: Type-II diabetes mellitus, BMI: Body mass index, TSH: Thyroid stimulating hormone, HbA1c: Hemoglobin A1c .

**Table-VII:** Logistic regression analysis for predicting elevated anti-TPO levels.

	B	S.E.	Exp(B)	p	95% CI.for Exp(B)
Constant	-3.776	1.598	0.023	0.018	
Type-I DM	1.582	0.561	4.862	0.005	1.619-14.604
Type-II DM	0.090	0.518	1.095	0.861	0.397-3.020
Gender (Women)	1.145	0.410	3.142	0.005	1.407-7.018
Age	0.014	0.016	1.014	0.382	0.983-1.045
BMI (kg/m2)	-0.010	0.031	0.990	0.747	0.932-1.052
TSH (mIU/ml)	0.271	0.151	1.312	0.072	0.976-1.762
HbA1c (%)	-0.024	0.087	0.976	0.782	0.823-1.158
Blood kreatinine	0.445	1.158	1.561	0.700	0.161-15.091

Cox & Snell R Square: 0.085, Nagelkerke R Square: 0.133. Abbreviations: Anti-TPO: Anti-thyroid peroxidase antibodies, Type-I DM: Type-I diabetes mellitus, Type-II DM: Type-II diabetes mellitus, BMI: Body mass index, TSH: Thyroid stimulating hormone, HbA1c: Hemoglobin A1c.

In predicting the factors influencing the changes in elevated anti-TG and anti-TPO levels, Type-I DM and women were found to besignificant (elevated anti-TG, Exp (B): 3.863, 2.543, p: 0.028; 0.035; elevated anti-TPO, Exp (B): 4.862, 3.142, p: 0.005, 0.005).

## DISCUSSION

Our study is one of the few that examines the levels of thyroid antibodies in - both women and men with Type-I and Type-II DM and in the control groups, all of whom have normal thyroid function. The results indicated that Type-I DM and women were predictive factors for elevated anti-TG and anti-TPO levels. Conversely, there was no significant difference in thyroid antibody levels were observed between patients with Type-II DM and those in the control group. Age, TSH, and HbA1c did not predict chronic thyroiditis.

In a cohort study reported by Vallianou et al. 256 adult patients with Type-I DM, a higher women ratio was observed in those with both Type-I diabetes and AIT, consistent with our study. 35.4% had AIT and elevated levels of both anti-TPO and anti-TG have been observed in 89% of patients with Type-I DM and AIT.^13^ In this study, 39 (37.9%) of Type-I DM had elevated anti-TG and anti-TPO levels. We identified 81 patients with elevated anti-TG and anti-TPO levels in all groups, of whom 39 (48.2%) had Type-I DM, resulting in a coexistence rate of both thyroid antibodies of 18 (46.2%) in Type-I DM. The higher rates of both antibodies in this study, conducted by Vallianou et al.^13^ may be attributed to the inclusion of individuals with thyroid disease, which differs from the results of this study.

In a study conducted by Chakarova et al. on 160 adults with Type-I DM, anti-TPO was detected in 22.5% of patients and anti-TG were elevated in 8.8% of patients. The incidence of AIT was reported to be higher in women than men, which is consistent with our study. However, thyroid antibodies were elevated in 24.4% of the cases, whereas in this study, the rate was 37.9%. Additionally, the fact that half of the patients had received a new diagnosis and the average duration of diabetes was 1.35 years may explain the lower rate.^14^ In this study, the median duration of diabetes was nine years.

In a study by Bilginer et al. involving 287 adults diagnosed with Type-I DM, 81.9% of the patients were euthyroid. Consistent with our study, thyroid antibodies in women were reported to be 42.3%, which is higher than that in men. Anti-TPO and anti-TG positivity rates were 34.8% and 19.9%, respectively. The proportion of patients who tested positive for thyroid antibodies was 36.2%. This rate was 37.9% in our study, similar to that reported by Bilginer et al.^15^ Hazzaa et al. assessed 76 individuals with Type-I diabetes with an average age of 15 years (range: 7-41 years). The anti-TPO positivity rate was 15.7%, with 83% women patients.^16^ In a study by Mohammedsaeed et al., involving 2258 children, adolescents, and adults, AIT was identified in 36.9% of the patients, with rates of 25.7% in adult women and 10.8% in adult men.^17^

In a study conducted by Schroner et al. with 79 euthyroid diabetic patients, chronic AIT was diagnosed in 40% (n = 8) of patients with Type-I DM, 50% (n = 6) of patients with latent autoimmune diabetes in adults (LADA), 43% (n = 20) of patients with Type-II DM, and 33% (n = 10) of the control group. In patients with Type-I DM, 30%, in the LADA subtype 50%, in patients with Type-II DM 25% and in the control group, 13% showed positivity for anti-TPO. The reported rates of anti-TG positivity were 0% in Type-I DM, 8% in LADA subtype, 6.4% in Type-II DM, and 3% in control, respectively.^18^ In the present study, unlike our study, an increased prevalence of chronic autoimmune thyroiditis was observed in patients with all types of diabetes mellitus. The small number of patients and regional factors may account for this difference.

In the study of Akbar and colleagues, including 74 patients with Type-II DM, 26 with LADA subtype, and 100 control subjects, thyroid dysfunction was detected in 7% of Type-II diabetic patients, 42% of LADA patients, and 7% of the control group. The prevalence of thyroid autoimmunity in the LADA, Type-II, and control groups was 27%, 4%, and 5%, respectively.^19^ In Akbar’s study, GAD65ab positivity was found in 26 of 100 patients with Type-II DM, with no sex-based evaluation.

Elebrashy et al., which included 62 female patients with Type-II DM. Among 34 euthyroid women diabetic patients, six (17.6%) had both antibodies negative, 11 (32.4%) had only anti-TPO positivity, three (8.8%) had only anti-TG-positivity, and 14 (41.2%) had both antibodies positive.^20^ In our study, 62 (80.5%) of the women patients with Type-II DM had both antibodies negative, 7 (9.1%) had only anti-TPO positive, two (2.6%) had only anti-TG-positivity, and 6 (7.8%) had both antibodies positive. The mean age in the study by Elebrashy et al was 44 years, whereas in our study, the median age was 56 years. Despite the age difference, the variation in the proportion of patients with both antibodies negative between the two studies is notable.

In the study reported by Elgazar and colleagues, 200 patients with Type-II DM, 11.1% of women with overt hypothyroidism, 57.2% were positive for thyroid antibodies, which were 42.9% positive for anti-TPO alone, 0% for anti-TG alone, and 14.3% positive for both antibodies. It has been found that anti-thyroid antibodies positivity is more common in women.^21^ In our study, women patients with Type-II DM were euthyroid, with an anti-TPO positivity rate of 9.1%, which was not significantly different from the control group. Elgazar’s study evaluated thyroiditis rates in subjects with thyroid dysfunction. In the present study, both the diabetes and control groups were found to be euthyroid. Research conducted on euthyroid Type-II diabetic patients has indicated considerable variation in thyroiditis rates, and there are limited studies examining thyroid autoimmunity and sex differences.

### Limitations

Our study has limitations, including its single-center design, absence of examination of patient follow-up data, lack of thyroid ultrasound data, and family history of thyroid diseases in the dataset. In addition, although the durations of diabetes are similar, differences in the average age of the study groups are also a limitation of our study.

## CONCLUSION

The present study determined that there is an association between Type-I diabetes mellitus and elevated levels of anti-TPO and anti-TG antibodies in women. Consequently, the assessment of thyroid antibodies may be recommended, particularly in women with Type-I diabetes mellitus, even in cases where the patient is euthyroid. However, in euthyroid men with Type-I diabetes and in both women and men with Type-II diabetes mellitus, where no significant difference in terms of thyroid antibodies was observed, an evaluation may not be necessary.
